# Cavernomatous Transformation of the Portal Vein in Crohn’s Disease: An Unexpected Radiologic Finding

**DOI:** 10.7759/cureus.109599

**Published:** 2026-05-25

**Authors:** Samia Obilat, Yahya Elharras, Houda Bennani, Kaoutar Imrani, Ittimade Nassar

**Affiliations:** 1 Radiology Department, Ibn Sina Hospital, Mohammed V University, Rabat, MAR

**Keywords:** cavernomatous transformation, computed tomography, crohn’s disease, portal vein thrombosis, splanchnic venous thrombosis

## Abstract

Crohn’s disease is associated with an increased risk of thromboembolic events due to chronic systemic inflammation and endothelial dysfunction. Portal vein thrombosis is a rare manifestation that may remain clinically silent and progress to cavernomatous transformation.

We report the case of a 32-year-old male patient with ileal Crohn’s disease in whom contrast-enhanced abdominal computed tomography (CT), performed for routine disease monitoring, revealed extensive chronic portal vein thrombosis with cavernomatous transformation.

This case highlights the important role of cross-sectional imaging in diagnosing chronic splanchnic venous thrombosis in Crohn’s disease, even in the absence of an acute inflammatory flare, and emphasizes the thrombotic mechanisms underlying inflammatory bowel disease.

## Introduction

Crohn’s disease is a chronic inflammatory bowel disease characterized by transmural intestinal inflammation and systemic immune activation. Beyond gastrointestinal involvement, patients with Crohn’s disease are at risk of extra-intestinal complications, among which thromboembolic events represent a significant cause of morbidity and mortality. The risk of venous thromboembolism in inflammatory bowel disease is estimated to be two to three times higher than in the general population [1,2].

While deep vein thrombosis and pulmonary embolism are the most frequently reported thrombotic manifestations, splanchnic venous thrombosis remains rare. Portal vein thrombosis is an uncommon but potentially severe complication that may remain asymptomatic or present with nonspecific symptoms, leading to delayed diagnosis. In chronic cases, portal vein thrombosis may progress to cavernomatous transformation, characterized by the development of periportal collateral venous networks and complications related to portal hypertension [3,4].

We report a radiological case of chronic portal vein thrombosis with cavernomatous transformation in a patient with Crohn’s disease, emphasizing the imaging findings and reviewing the thrombotic mechanisms associated with inflammatory bowel disease.

## Case presentation

A 32-year-old male patient with a known history of ileal Crohn’s disease was referred for abdominal computed tomography (CT). The disease had been previously managed surgically, with ileal resection and ileo-ileal anastomosis performed in 2023.

At the time of imaging, there was no clinical or biological evidence of an acute inflammatory flare of Crohn’s disease. The CT examination was performed for routine disease monitoring in the absence of abdominal pain or symptoms suggestive of portal hypertension.

No signs of acute inflammatory flare were observed. However, a segmental ileal wall thickening, likely reflecting chronic changes related to Crohn’s disease, was identified.

A contrast-enhanced abdominal CT was performed, revealing the absence of opacification of the main portal vein, with thrombosis extending to its bifurcation and associated cavernomatous transformation, characterized by multiple serpiginous periportal venous collaterals at the hepatic hilum (Figure 1).

**Figure 1:**
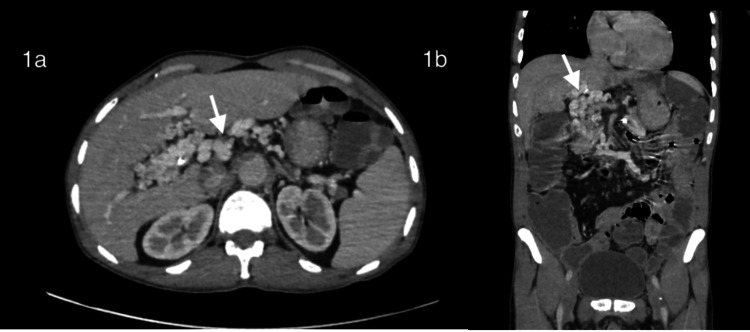
Contrast-enhanced abdominal CT images demonstrating cavernomatous transformation of the portal vein. (A) Axial portal venous phase image showing the absence of portal vein opacification with serpiginous periportal collaterals (arrow). (B) Coronal reformatted image illustrating the extent of the cavernomatous transformation (arrow).

The thrombosis extended to the superior mesenteric vein and the spleno-mesenteric trunk (Figure 2).

**Figure 2:**
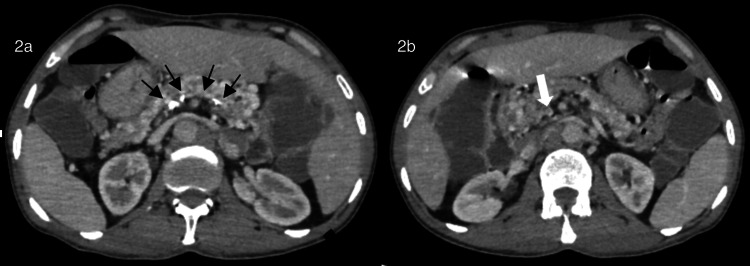
Contrast-enhanced abdominal CT images showing extension of the portal vein thrombosis to the spleno-mesenteric trunk and superior mesenteric vein. (A) Axial image showing thrombosis of the spleno-mesenteric trunk (black arrows). (B) Axial image showing extension to the superior mesenteric vein (white arrow).

Numerous porto-systemic collateral pathways, including perigastric and gastro-splenic shunts, were identified, consistent with a chronic splanchnic venous thrombosis (Figure 3).

**Figure 3:**
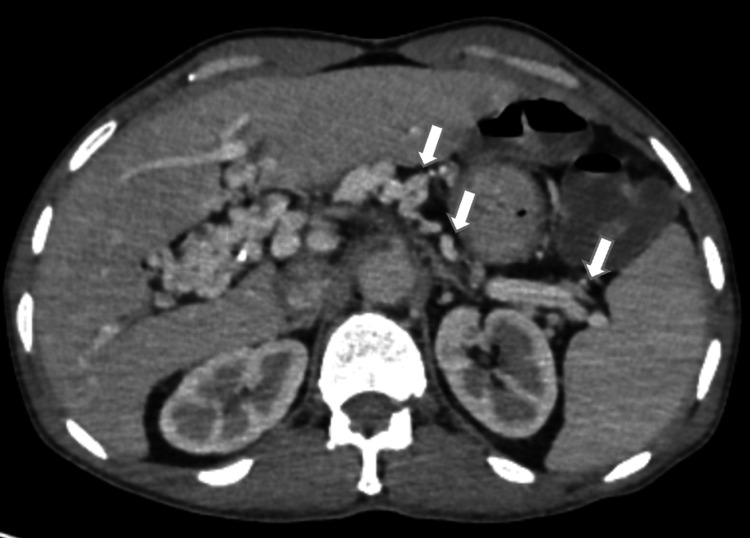
Contrast-enhanced abdominal CT image demonstrating extensive porto-systemic collateral circulation. Axial image showing multiple porto-systemic collateral vessels (arrows), consistent with chronic splanchnic venous thrombosis.

No ascites or focal hepatic lesion was identified.

Figure 4 demonstrates a segmental ileal wall thickening, consistent with chronic inflammatory changes related to Crohn’s disease.

**Figure 4:**
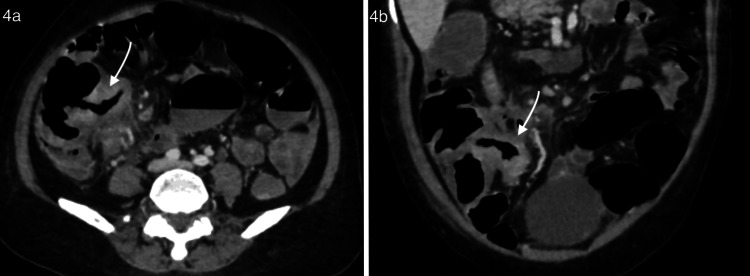
Contrast-enhanced abdominal CT images showing segmental ileal wall thickening. (A) Axial image showing segmental circumferential ileal wall thickening (arrow). (B) Coronal reformatted image confirming the segmental ileal thickening (arrow).

## Discussion

Patients with Crohn’s disease are at increased risk of venous thromboembolism compared to the general population, due to chronic systemic inflammation and a persistent prothrombotic state [1,2].

Portal vein thrombosis represents a rare but clinically significant manifestation of thromboembolic disease in this context [3-5]. It is often asymptomatic and may be discovered incidentally during imaging performed for other indications. In chronic cases, it may evolve toward cavernomatous transformation, characterized by the development of periportal collateral venous networks and portal hypertension-related complications [3,4].

Several risk factors contribute to the occurrence of portal vein thrombosis in patients with Crohn’s disease. In addition to systemic inflammation, abdominal surgery is a major contributing factor, promoting thrombosis through endothelial injury, local inflammation, and venous stasis [5]. Other contributing factors include dehydration, immobilization, and corticosteroid therapy.

The pathophysiology of thromboembolic events in inflammatory bowel disease is multifactorial. Chronic inflammation induces activation of the coagulation cascade, platelet activation, impaired fibrinolysis, and endothelial dysfunction, resulting in a sustained hypercoagulable state even in the absence of an acute inflammatory flare [6].

From a radiological perspective, cross-sectional imaging plays a pivotal role in the diagnosis, characterization, and follow-up of portal vein thrombosis. Contrast-enhanced computed tomography allows accurate assessment of the extent of thrombosis, differentiation between acute and chronic stages, and identification of cavernomatous transformation. Typical imaging findings include the absence of portal vein opacification, replacement by serpiginous periportal collateral vessels, and the presence of porto-systemic shunts, which are characteristic of cavernomatous transformation [7]. Imaging also enables evaluation of portal hypertension-related complications and detection of associated bowel abnormalities related to Crohn’s disease. In our case, segmental ileal wall thickening supports the chronic nature of the underlying inflammatory condition.

Portal vein thrombosis and cavernomatous transformation in inflammatory bowel disease are most commonly described in patients with active inflammatory disease, postoperative status, or additional thrombotic risk factors. Similar cases of extensive splanchnic venous thrombosis associated with Crohn’s disease have also been reported in the literature [5,8]. In contrast, our case illustrates the incidental radiological detection of extensive chronic portal vein thrombosis with cavernomatous transformation in a clinically stable patient with Crohn’s disease, emphasizing the importance of systematic evaluation of the portal venous system during routine abdominal imaging.

The management of portal vein thrombosis in inflammatory bowel disease relies primarily on anticoagulation therapy, which aims to prevent thrombus extension, promote recanalization, and reduce the risk of recurrence [4]. According to current guidelines, treatment should be individualized based on the extent of thrombosis and patient-specific risk factors [9]. In chronic cases with cavernomatous transformation, complete recanalization is unlikely, and management focuses on preventing and treating complications of portal hypertension. Interventional radiological procedures, particularly transjugular intrahepatic portosystemic shunt (TIPS), may be considered in selected patients and represent an important therapeutic option in this setting [7,9].

The prognosis depends on the extent of thrombosis and the development of complications. Early diagnosis is associated with improved outcomes, whereas chronic forms may lead to significant morbidity, particularly due to gastrointestinal bleeding. Long-term follow-up is therefore essential.

This case highlights the central role of imaging in detecting clinically silent portal vein thrombosis and its complications in patients with Crohn’s disease, even in the absence of an acute inflammatory flare, and underscores the importance of systematic evaluation of the portal venous system in this population.

## Conclusions

Chronic portal vein thrombosis with cavernomatous transformation is a rare but clinically significant complication of Crohn’s disease. This case illustrates that splanchnic venous thrombosis may remain clinically silent and progress to chronic forms, particularly in patients with a history of abdominal surgery. Radiologists should be aware of this entity and systematically assess the portal venous system in patients with inflammatory bowel disease, even in the absence of an acute inflammatory flare.
